# Physiological Sensors Equipped in Wearable Devices for Management of Long COVID Persisting Symptoms: Scoping Review

**DOI:** 10.2196/69506

**Published:** 2025-03-26

**Authors:** Shikha Kukreti, Meng-Ting Lu, Chun-Yin Yeh, Nai-Ying Ko

**Affiliations:** 1 Department of Nursing, College of Medicine National Cheng Kung University Tainan City Taiwan; 2 Deparment of Public Health, College of Medicine National Cheng Kung University Tainan City Taiwan; 3 Department of Computer Science and Information Engineering National Cheng Kung University Tainan City Taiwan; 4 International Doctoral Program in Nursing, Deaprtment of Nursing, College of Medicine National Cheng Kung University Tainan City Taiwan

**Keywords:** wearable devices, long COVID, physiological sensors, review, COVID, COVID-19

## Abstract

**Background:**

Wearable technology has evolved in managing COVID-19, offering early monitoring of key physiological parameters. However, the role of wearables in tracking and managing long COVID is less understood and requires further exploration of their potential.

**Objective:**

This review assessed the application and effectiveness of wearable devices in managing long COVID symptoms, focusing on commonly used sensors and their potential for improving long-term patient care.

**Methods:**

A literature search was conducted across databases including PubMed, Embase, Web of Science, and Cochrane Central, adhering to PRISMA-ScR (Preferred Reporting Items for Systematic Reviews and Meta-Analyses extension for Scoping Reviews) reporting guidelines. The search was updated regularly throughout 2024. Abstract and full-text screening and selection were facilitated using Rayyan software developed by Qatar Computing Research Institute. Quality appraisal was conducted using the Joanna Briggs Institute (JBI) critical appraisal tool to ensure the methodological rigor of the included studies. Data were extracted on study characteristics, wearable devices, sensors used, and monitored physiological parameters, and the results were synthesized in a narrative format.

**Results:**

A total of 1186 articles were identified, and after duplicate removal and screening, 15 studies were initially included, with 11 studies meeting the criteria for final data synthesis. The included studies varied in design, ranging from observational to interventional trials, and involved sample sizes from 3 to 17,667 participants across different countries. In total, 10 different wearable devices were used to monitor long COVID symptoms, capturing key metrics such as heart rate variability, body temperature, sleep, and physical activity. Smartwatches were the most used wearable devices and fitness trackers with electrocardiography and photoplethysmography sensors were used to monitor heart rate, oxygen saturation, and respiratory rate. Of the 10 devices, 4 were Food and Drug Administration–approved, emphasizing the reliability and validation of the physiological data collected. Studies were primarily conducted in the United States and Europe, reflecting significant regional research interest in wearable technology for long COVID management.

**Conclusions:**

This review highlights the potential of wearable technology in providing continuous and personalized monitoring for long COVID patients. Although wearables show promise in tracking persistent symptoms, further research is needed to improve usability, validate long-term efficacy, and enhance patient engagement.

## Introduction

Wearable devices have emerged as essential tools in the detection and management of COVID-19 due to their ability to continuously monitor key physiological metrics such as heart rate, oxygen saturation, and body temperature in real time [1,2]. These devices provide a proactive approach to identifying early signs of COVID-19, enabling timely interventions even before severe symptoms appear. Unlike acute COVID-19, where symptoms typically manifest and resolve within a few weeks, long COVID involves persistent and varied symptoms that can last for months or even years after the initial infection [3]. This complexity requires not only the ability to detect acute symptoms but also to monitor and manage chronic and fluctuating conditions over extended periods. Long COVID is a multifaceted condition that requires not only a deep understanding of its complex pathophysiology but also the integration of advanced digital technologies like wearables to effectively monitor, analyze, and manage its persistent and fluctuating symptoms over time. For patients with long COVID, wearable technology offers invaluable insight into persistent and fluctuating symptoms, helping to track and manage the condition over extended periods [4]. Despite their potential, there is still a need to refine and enhance the effectiveness of wearable technology, ensuring consistency in how different devices capture and analyze data for long COVID’s complex health effects.

Although a few studies have investigated the use of wearable devices for long COVID detecting symptoms, findings have varied due to differences in device types, sensor configurations, and analytical methods [5,6]. This variation underscores the need for a comprehensive review to synthesize existing knowledge, evaluate the effectiveness of different wearable technologies, and identify best practices for their application in long COVID management. A thorough review can help bridge the gaps in this research and guide future innovations in wearable technology for more effective monitoring and management of long COVID.

Wearable devices equipped with sensors for monitoring heart rate, respiratory rate, oxygen saturation, and body temperature have shown promise in detecting anomalies associated with COVID-19 [7]. Previous studies have explored the role of wearable devices in the detection of COVID-19 symptoms, demonstrating their potential to provide early warnings and facilitate timely interventions [7-9]. Research has demonstrated that changes in these metrics can indicate the onset of infection before severe symptoms manifest [10]. However, while these devices have been effective in detecting acute COVID-19 symptoms, managing long COVID presents a more complex challenge, as it involves tracking persistent, fluctuating symptoms over extended periods, requiring continuous and adaptable monitoring strategies.

Understanding physiological sensors equipped in wearable devices is crucial because it enables continuous and precise monitoring of post-COVID complex symptoms, providing insights into their patterns and severity over time. Effective management of long COVID relies on identifying which sensors are most used and how they perform in capturing relevant physiological data. Current literature reveals a variety of sensors used in wearable devices, such as those for monitoring heart rate variability, oxygen saturation, and respiratory rate [11]. Given the urgency to address long COVID effectively, a comprehensive scoping review is essential to consolidate existing research, evaluate the efficacy of different sensors, and inform the development of more targeted and effective monitoring solutions. The primary objective of this scoping review is to evaluate and summarize existing research on the application of wearable technology for monitoring and managing persistent long COVID symptoms. This review seeks to identify the various wearable devices and sensors used, the specific physiological parameters they monitor, and the impact of this monitoring on the management of long COVID.

## Methods

### Overview

This scoping review was conducted in adherence with the PRISMA-ScR (Preferred Reporting Items for Systematic Reviews and Meta-Analyses extension for Scoping Reviews) reporting guidelines [12]. Ethical approval was not required for this Scoping review. Refer to Multimedia Appendix 1 for the PRISMA checklist.

### Search Strategy and Selection Criteria

A comprehensive literature search was conducted across electronic databases, including PubMed (MEDLINE), Embase, Cochrane Central Register of Controlled Trials (known as CENTRAL), and the international clinical trials registry platform. An additional search for datasets was conducted by SK using Google Scholar. The reference lists of included articles and reviews were manually searched to identify additional relevant studies for full-text screening. To ensure as current a review as possible, we repeated the above searches on June 15, 2024, and July 10, 2024, and again on November 15, 2024, before final analysis. The search strategy combined keywords related to long COVID (eg, “Long COVID,” “Post-Acute Sequelae of SARS-CoV-2 Infection,” “PASC”), wearable technology (eg, “wearable devices,” “smartwatches,” “fitness trackers”), and physiological monitoring (eg, “heart rate variability,” “blood oxygen saturation,” “respiratory rate;” Multimedia Appendix 2).

### Selection Process and Data Extraction

This scoping review focused on studies that involved the use of wearable technology for monitoring or managing long COVID symptoms, specifically those published in peer-reviewed journals. It included research that reported on specific physiological parameters monitored by wearable devices, such as heart rate, respiratory rate, and oxygen saturation. The review excluded studies that did not specifically focus on long COVID, those that did not involve wearable technology, and those that were not available in full text, including review articles, conference abstracts, or incomplete articles. The initial abstract screening was conducted using Rayyan [13], a web-based tool for systematic reviews. The title and abstracts were independently reviewed by 2 reviewers (SK and MTL), and a third reviewer (NYK or CYL) acted as an arbitrator in case of disagreements between the 2 other reviewers.

Data were extracted from the included studies using a JBI standardized form. Information on study characteristics (eg, author, year, country), type of wearable device used, sensors used, physiological parameters monitored, and the outcomes related to long COVID management were recorded. A narrative synthesis was conducted to summarize the findings, with a focus on the types of wearable devices and sensors used, the physiological parameters monitored, and the impact on long COVID symptom management.

## Results

### Overview

**Figure 1:**
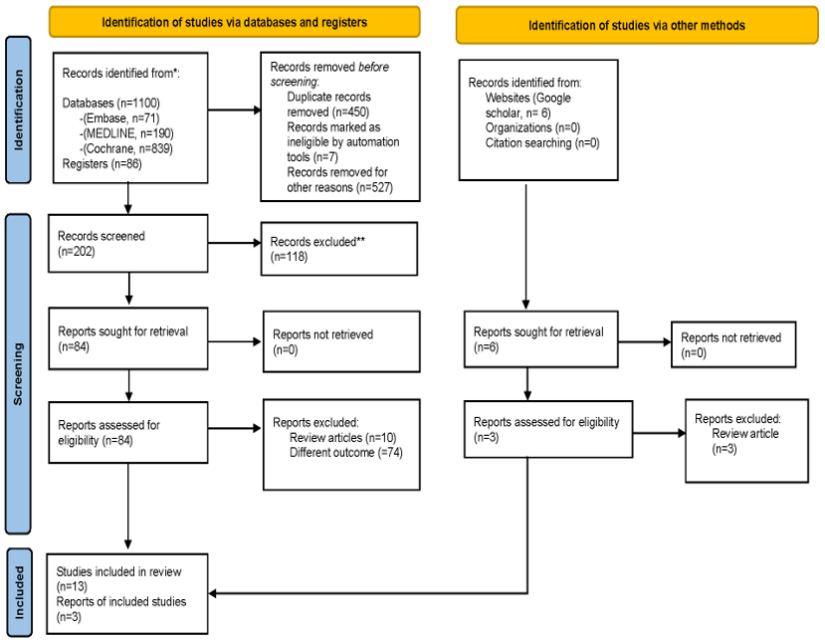
Preferred Reporting Items for Systematic reviews and Meta-Analyses (PRISMA) 2020 flow diagram showing identification of studies from search databases.

A total of 1186 articles were identified from 3 databases, 1 registry, and Google Scholar. Following the removal of duplicates and the initial eligibility screening, 16 studies were included in the review (Figure 1). These 16 studies underwent a JBI quality assessment by the 2 authors, and out of these, only 11 met the criteria for data synthesis. Therefore, a total of 11 studies were included in the final review.

### Study Characteristics

A total of 11 studies met the inclusion criteria and were included in the review [5,6,14-22]. These studies varied in design, ranging from observational studies to interventional trials, and were conducted across different countries (Table 1). The studies’ sample sizes ranged widely from as few as 3 participants [20] to as many as 17,667 participants [21].

**Table 1:** Characteristics of the included 11 studies.

Number	Author and year	Country and region	Study design	Sample size	Wearable technology	Regulatory status
1	Mekhael et al (2022) [5]	New Orleans, United States	Prospective observational study	710	Biostrap	FDA-approved^a^
2	Lonini et al (2021) [14]	Illinois, United States	Pilot study	28	Suprasternal notch device	N/A^b^
3	Mekhael et al (2024) [19]	United States	Observational	122	Biostrap	FDA-approved
4	Xue et al (2022) [15]	Ireland	Cross-sectional	108	Shimmer device, Finapres New Observation and Verification Apparatus	Published study by Schutte et al, 2004
5	Laguarta-Val et al (2024) [16]	Madrid, Spain	A nonrandomized parallel controlled trial	29	Polar Ignite 2 device	N/A
6	Corrado et al (2024) [17]	United Kingdom	Pilot	13	Polar H10 chest Strap or Fitbit Charge 5 smartwatch	FDA-approved (Fitbit)
7	Corrêa et al (2023) [18]	Brazil	Cross-sectional	73	Samsung mobile phone	N/A
8	Romaszko-Wojtowicz et al (2022) [20]	Poland	Case study	3	Aidmed	American National Standard for Ambulatory Electrocardiographs
9	Kerling et al (2024) [6]	Germany	Prospective, randomized, parallel-group	62	Garmin	FDA-approved
10	Stewart et al (2024) [21]	United Kingdom	Longitudinal case-control	17,667	Fitbit	FDA-approved
11	Radin et al (2024) [22]	United States	Prospective observational study	553	Fitbit	FDA-approved

^a^FDA: Food and Drug Administration.

^b^Not applicable.

### Wearable Devices and Sensors

The wearable devices identified in the studies included a mix of commercially available devices (eg, smartwatches, fitness trackers) and research-specific devices designed for health monitoring. In total, 10 different wearable devices were used to monitor long COVID symptoms, capturing key metrics such as heart rate variability (HRV), body temperature, sleep, and physical activity, providing a comprehensive understanding of the physiological effects of the condition. The most used devices were smartwatches (eg, Biostrap, Fitbit, Garmin) and fitness trackers, which are widely accessible and user-friendly.

In the included studies, seven different types of sensors were used, including photoplethysmography (PPG), electrocardiography (ECG), accelerometers, temperature sensors, gyroscopes, near-infrared spectroscopy, and electromyography (Table 2). These sensors were used across multiple devices to monitor key physiological metrics such as heart rate, heart rate variability, peripheral capillary oxygen saturation (SpO_2_), respiratory rate, temperature, physical activity, and sleep-related movements [5,6,14-22]. Sensors integrated into these devices were primarily focused on monitoring cardiovascular and respiratory parameters.

**Table 2:** Study wearable device details of included 11 studies.

Number	Author	Sensor	Physiological characteristics
1	Mekhael et al (2022) [5]	PPG^a^ Accelerometer	Heart rate Heart rate variability Respiratory rate SpO2^b^ Movement
2	Lonini et al (2021) [14]	Adam sensor	Body motions Vibrations induced by sounds produced by heartbeats, coughing, or breathing
3	Mekhael et al (2024) [19]	PPG Accelerometer	Heart rate Heart rate variability Respiratory rate SpO2 Movement
4	Xue et al (2022) [15]	Digital artery photoplethysmography Near-infrared spectroscopy Electromyography	Heart rate Heart rate variability SpO2 Fatigue
5	Laguarta-Val et al (2024) [16]	ECG^c^ temperature accelerometer	Heart rate Temperature Physical activity
6	Corrado et al (2024) [17]	ECG PPG Accelerometer or gyroscope	Heart rate variability Heart rate Sleep rotation movement
7	Corrêa et al (2023) [18]	Accelerometers Gyroscopes (inertia sensors)	Physical activity or mobility
8	Romaszko-Wojtowicz et al (2022) [20]	ECG Temperature PPG	Heart rate Temperature Respiratory rate
9	Kerling et al (2024) [6]	ECG Accelerometer	Physical activity Heart rate Sleep
10	Stewart et al (2024) [21]	ECG Accelerometers	Heart rate Heart rate variability Physical activity and step count
11	Radin et al (2024) [22]	ECG Accelerometers	Resting heart rate Physical activity and step count Sleep

^a^PPG: photoplethysmography.

^b^SpO_2_: peripheral capillary oxygen saturation.

^c^ECG: electrocardiography.

ECG was used in 6 out of the 10 studies [14,16,22] and was used to monitor heart rate, and heart rate variability, and to detect arrhythmias and other cardiac abnormalities.

PPG was used in 3 studies [5,17,19] and was also used to measure heart rate, SpO₂ levels, and respiratory rate.

Accelerometers were integrated in 6 studies [6,17] and used to monitor physical activity, sleep patterns, and mobility. They are critical for measuring movements and providing insights into daily activity levels and sleep quality.

Some studies reported the use of more advanced sensors, such as Adam sensors [14] which are used to monitor body motion as it works on vibrations.

### Food and Drug Administration Approval and Device Validation

Devices like Fitbit [17,21,22], Polar Ignite [16,17], Garmin [17], and Biostrap [5,19] got Food and Drug Administration (FDA) clearance for ECG functionalities, ensuring regulatory oversight and validation of the accuracy, safety, and reliability of the data collected (Table 1). In addition, the medical device Aidmed is approved by the American National Standard for Ambulatory Electrocardiographs.

### Physiological Parameters Monitored

The physiological parameters monitored by these wearable devices included heart rate, heart rate variability, blood oxygen saturation, respiratory rate, skin temperature, and physical activity levels (Figure S1 in Multimedia Appendix 2). These parameters were chosen for their relevance to the symptoms experienced by individuals with long COVID, such as fatigue, autonomic dysfunction, and respiratory distress.

### Country of Origin and Regional Focus

The studies were predominantly conducted in the United States and Europe.

Five studies originated in the United States, reflecting a significant research interest in wearable technology for long COVID in this region. The studies by Mekhael et al [5], Radin et al [22], and Lonini et al [14] demonstrated extensive use of wearable devices like Biostrap (Sontakey), Fitbit (Google), and the Suprasternal notch device (Sonica Health**)** [19].

European studies (United Kingdom, Spain, Poland, Germany, and Ireland) were also included, indicating a broad European engagement in the field. The study by Xue et al [15] in Ireland used the Shimmer device (Shimmer Sensing) and Finapres NOVA (Finapres Medical Systems), while the United Kingdom–based study by Corrado et al [17] used the Polar H10 chest strap (manufactured by Polar Electro) and Fitbit Charge 5 smartwatch. Similarly, the study by Kerling et al [6] in Germany used a Garmin device (Garmin Ltd).

### Measurement Approaches in Long COVID Studies

Devices like Biostrap and Fitbit tracked sleep stages, HRV, and physical activity, helping to identify sleep disruptions and reduced physical endurance in patients (Multimedia Appendix 3). Tools such as the Suprasternal notch device and Shimmer captured critical data on altered respiration, cough frequency, and neurocardiovascular responses, shedding light on the multisystem impacts of long COVID. Interventions using devices like the Polar H10 and Garmin provided insights into HRV biofeedback and energy expenditure, aiding rehabilitation and symptom relief. Overall, these devices offer valuable tools for understanding and managing long COVID symptoms, enabling tailored care and long-term monitoring for affected individuals.

## Discussion

### Principal Findings

This scoping review offers a comprehensive evaluation of the existing literature on the use of wearable health devices for monitoring and managing long COVID symptoms. The findings highlight the potential of wearable devices to revolutionize the management of this complex and persistent of long COVID symptoms by continuous physiological monitoring. This review identifies a wide range of wearable devices used in published studies, including commercially available smartwatches, fitness trackers, and research-specific medical devices. The use of 10 different wearable devices in detecting long COVID symptoms highlights the potential of these technologies in early symptom identification [5,6,14-22]. Sensors such as PPG, ECG, and accelerometers were commonly used to monitor cardiovascular and respiratory functions, reflecting the prevalence of these symptoms in long COVID patients. These devices continuously track key physiological parameters like heart rate, HRV, SpO_2_, and sleep patterns, allowing for the early detection of subtle health changes and proactive management of symptoms. The widespread use of accelerometers also emphasizes the growing importance of physical activity and sleep monitoring in managing chronic conditions like long COVID.

One notable finding in this review is that only a limited number of wearable devices have received FDA approval for specific health monitoring features. For instance, Fitbit’s ECG app, cleared by the FDA in 2020, is accessible on models such as the Sense, Sense 2, and Charge 5 [23]. Garmin has also secured FDA clearance for its ECG app, now available on compatible models like the Venu 2 Plus and Epix Pro series [24]. The inclusion of FDA-approved devices brings a level of regulatory oversight, which is critical in clinical settings where wearable data may influence patient care decisions. FDA-approved wearables are validated for safety and effectiveness, offering health care providers more confidence in the data collected, especially when managing complex conditions like long COVID. In contrast, studies that do not emphasize the use of FDA-approved devices may face challenges in translating their findings into clinical practice. Comparing these studies underscores the need for standardized protocols and further validation studies to assess the long-term efficacy of wearable devices, ensuring their integration into routine health care for monitoring and managing long COVID symptoms.

This review also highlights issues related to user adherence and engagement. The success of wearable technology in managing chronic conditions like long COVID depends not only on the accuracy of the data collected but also on the willingness of users to consistently wear the devices and engage with the data. A few studies reported issues with user adherence, which could be attributed to factors such as device comfort, battery life, and the user interface of the accompanying apps [19,25]. Despite advancements in wearable technology, a significant gap exists in ensuring the seamless, secure, and standardized handling of the vast amounts of personal health data collected by these devices. In agreement with Smith et al [26], data interoperability and secure sharing across platforms, underscore the need for improved systems for data management and privacy to effectively integrate wearable technologies into clinical practice [26]. These issues highlight the need for more user-centered design approaches in the development of wearable devices, ensuring that they are not only functional but also comfortable and easy to use over extended periods.

In terms of the geographic distribution of the studies, the majority were conducted in the United States [5,14,19] and Europe [15-17,20,21], reflecting a significant research interest in these regions. This regional focus may be influenced by the availability of resources and funding for research on wearable technology, as well as the higher prevalence of long COVID in these areas due to the earlier and more widespread outbreaks of COVID-19. However, there is a need for more research in other regions, particularly in low- and middle-income countries, where the burden of long COVID may be significant but less well-documented. Furthermore, this review also found that while wearable devices offer valuable insights, the complexity and vast symptomatology of long COVID remain underexplored. Included studies focused on specific symptoms, with limited attention to common issues like fatigue, which is less frequently measured by these devices. This highlights the limitations of wearable technology in addressing the full spectrum of long COVID symptoms, underscoring the need for more comprehensive research.

This review provides a distinct perspective from typical COVID-19 wearable device studies by focusing on the long-term management of long COVID, rather than acute symptom detection. The key difference lies in the chronic nature of long COVID, where persistent and fluctuating symptoms, such as fatigue, cardiovascular issues, and sleep disturbances, require continuous, long-term monitoring. In contrast, COVID-19 reviews often emphasize short-term monitoring for acute symptoms, such as changes in respiratory rate and oxygen saturation, during the active phase of infection. Moreover, this review addresses unique challenges like user adherence and device validation for chronic use, which are less of a concern in acute COVID-19 monitoring.

There are several limitations to this review that must be acknowledged. One key limitation is the reliance on small sample sizes in some studies, which may reduce the statistical power and the ability to draw broader conclusions. In addition, the use of self-reported data in certain studies could introduce bias and affect the accuracy of the findings. Another limitation is the study design, with most studies lacking a longitudinal approach, which is crucial for assessing the long-term effectiveness of wearable technology in tracking long COVID symptoms. Furthermore, the number of studies available for review remains limited, highlighting the urgent need for more high-quality research to address the evolving challenges of long COVID management through wearable technology.

### Conclusion

This review highlights the significant potential of wearable technology in managing long COVID by leveraging sensors like ECG, PPG, and accelerometers to monitor crucial physiological metrics such as heart rate, oxygen saturation, and physical activity. Wearable devices provide real-time insights into persistent and fluctuating symptoms, offering a more personalized approach to patient care. The advancements in device accuracy and the increasing availability of FDA-approved options further reinforce their importance in clinical use. Furthermore, enhancing the usability of these devices to improve user engagement will enable broader adoption and sustained monitoring over extended periods. Ultimately, wearable technology stands out as a promising tool to support individualized care and improve outcomes for long COVID patients, shaping the future of health care monitoring.

### Future Implications and Requirements

For wearable technology to fully realize its potential in managing long COVID, several future implications are required. Standardization of protocols for sensor use and data interpretation is critical. This will allow for greater consistency across studies and ensure that health care providers can rely on the data generated by wearable devices. Wearability plays a crucial role in adherence, and future innovations should focus on compact, lightweight designs and hypoallergenic materials to enhance comfort and minimize user burden. Equally important is ensuring that sensors are accurate, durable, and capable of sustained performance in diverse real-world conditions. User-centered design is essential for improving adherence to wearable devices, ensuring that they are comfortable, easy to use, and integrated seamlessly into patients’ lives. Longitudinal studies are also needed to assess the sustained benefits of wearable technology in managing long COVID symptoms and improving health outcomes over time.

Building on the current research, the future applications of wearable health devices extend beyond long COVID management to other infectious diseases, such as influenza or emerging infectious diseases with similar symptomatology. Wearable devices that continuously monitor vital signs such as body temperature, heart rate, and respiratory functions could facilitate early illness detection and proactive management by identifying subtle physiological changes indicative of infection. Such applications are particularly relevant for influenza, where symptoms frequently overlap with long COVID, including fatigue, respiratory issues, and fever. In addition, integrating wearable data into public health systems could provide real-time insights into disease trends and hotspots, supporting more responsive and informed health interventions. Future efforts should also explore the integration of advanced data analytics and artificial intelligence to interpret sensor data more effectively, paving the way for personalized health care solutions.

This review illustrates that wearables have the potential to reshape not only individual patient care but also population-level disease management, positioning them as vital tools in addressing both chronic and acute public health challenges. Overall, to fully integrate wearable devices into long COVID management, a concerted effort is required to address these challenges. By developing standardized methods, improving device usability, and expanding research across diverse populations, wearable technology could become a cornerstone in the effective long-term management of long COVID.

## Appendix

Multimedia Appendix 1 PRISMA extension for scoping reviews checklist (PRISMA-ScR).

Multimedia Appendix 2 Literature search for conducting scoping review: documentation form.

Multimedia Appendix 3 Study outcomes and results.

## Abbreviations

ECG: electrocardiography
FDA: Food and Drug Administration
HRV: heart rate variability
JBI: Joanna Briggs Institute
PPG: photoplethysmography
PRISMA-ScR: Preferred Reporting Items for Systematic Reviews and Meta-Analyses extension for Scoping Reviews
SpO2: peripheral capillary oxygen saturation
