# Supplementary material for: Physiological Sensors Equipped in Wearable Devices for Management of Long COVID Persisting Symptoms: Scoping Review
Source: J Med Internet Res. 2025 Mar 26;27:e69506. doi: 10.2196/69506 (PMC11982746; doi:10.2196/69506)
Supplement: Multimedia Appendix 2 [file jmir_v27i1e69506_app2.docx]

**Literature Search for Conducting Scoping Review: Documentation Form**

**Table S1. Question**

| **Question:** | "How can physiological sensing through wearable technology be used to management of Long COVID persisting Symptoms?" |
| --- | --- |
| **P**opulation | COVID-19 patient with persistent symptom |
| **Concept** | Physiological sensing through wearable technology |
| **C**ontext | Management of Long COVID |

**Table S2. Search Terms**

|  | English Synonyms | **Emtree / MeSH/ Cochrane Library**  Controlled Vocabulary |
| --- | --- | --- |
| P | **'COVID long hauler' OR 'COVID-19 long-hauler' OR 'chronic COVID syndrome' OR 'long COVID' OR 'post COVID-19 fatigue' OR 'post COVID syndrome' OR 'post COVID condition'** | **long COVID**  **Post-Acute COVID-19 Syndrome**  **Long COVID** |
| **C** | **'wearable devices' OR 'wearable sensor' OR 'wearable device' OR 'biosensor' OR 'body sensor network' OR 'bsn' OR 'biomedical sensor' OR 'iot' OR 'wireless wearable technology' OR 'wireless wearable' OR 'wearable electronic devices' OR 'accelerometer' OR 'activity tracker' OR 'pedometer' OR 'sensor' OR 'smartphone'** | **wearable device**  **Wearable Electronic Devices**    **Wearable device** |
| **C/O** | **'monitor OR 'physiological monitor*' OR 'vital sign monitor' OR 'monitoring' OR 'disease management'** | **Monitoring, disease management**    **Disease Management**  **Monitoring, disease management** |

**Table S3. Searched Databases**

| Database | Date searched |
| --- | --- |
|  |  |
| 1. Embase | 71 |
| 1. PUBMED | 190 |
| 1. CENTRAL | 925 |
| 5)Additional sources  (Google scholar) | 7 |

**Table S4. Search Strategy**

| Database | # | Search syntax |
| --- | --- | --- |
| 1. **Embase** | 1 | **'covid long hauler' OR 'covid-19 long-hauler' OR 'chronic covid syndrome' OR 'long covid' OR 'post covid-19 fatigue' OR 'post covid syndrome' OR 'post covid condition' OR 'post infectious syndrome':ti,ab,kw,de** |
|  | 2 | 'long COVID'/exp OR 'post infectious syndrome'/exp |
|  | 3 | **'wearable devices' OR 'wearable sensor' OR 'wearable device' OR 'biosensor' OR 'body sensor network' OR 'bsn' OR 'biomedical sensor' OR 'iot' OR 'wireless wearable technology' OR 'wireless wearable' OR 'wearable electronic devices' OR 'accelerometer' OR 'activity tracker' OR 'pedometer' OR 'sensor' OR 'smartphone'**:ti,ab,kw,de |
|  | 4 | ' wearable device'/exp |
|  | 5 | **'monitor OR 'physiological monitor*' OR 'vital sign monitor' OR 'monitoring' OR 'disease management'**:ti,ab,kw,de |
|  | 6 | 'Monitoring'/exp Or 'disease management'/exp |
|  | 7 | **(#1 OR #2) AND (#3 OR #4) AND (#5 OR #6)**  **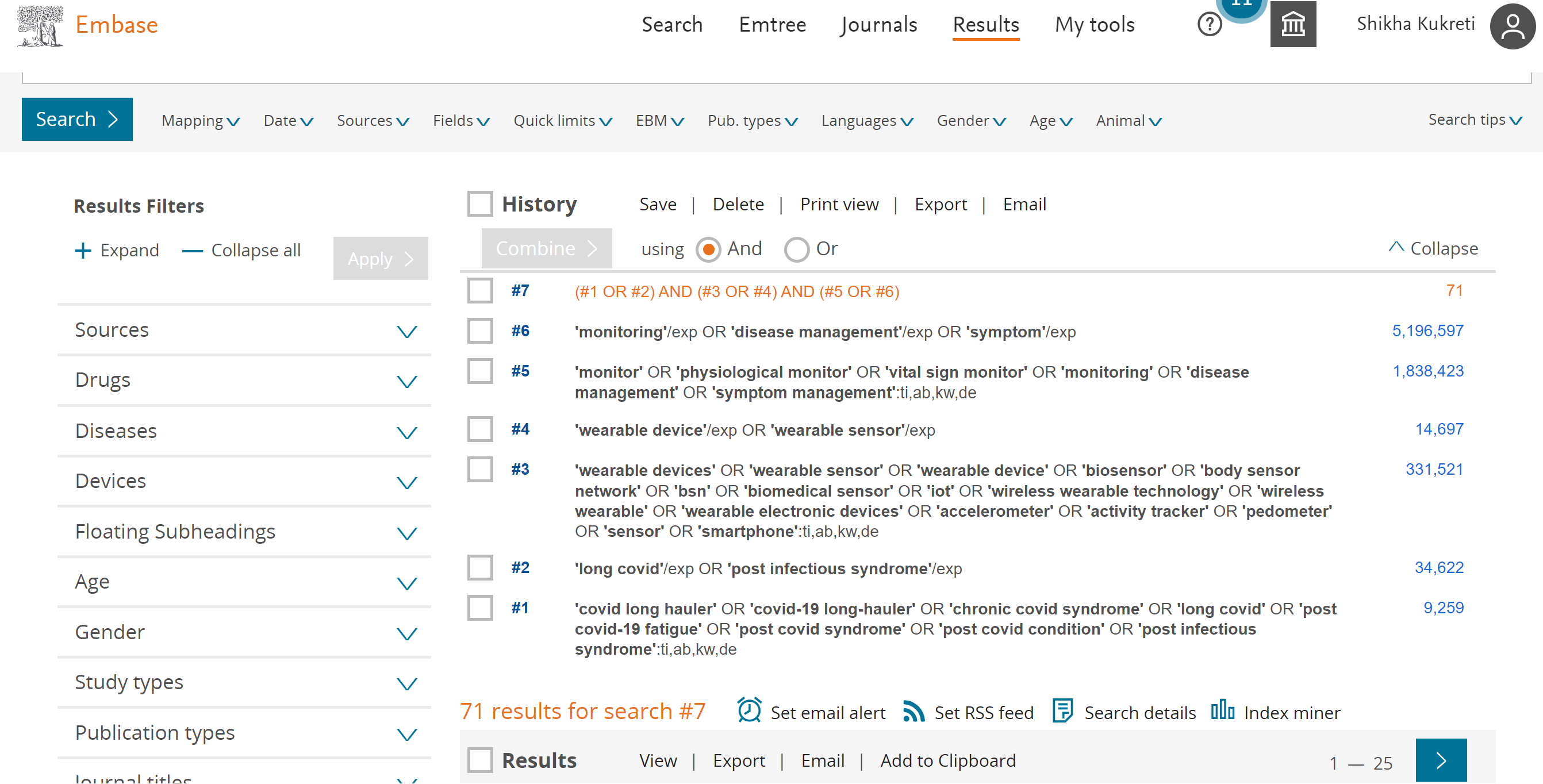** |
| **PubMed** | 1 | **'COVID long hauler' OR 'COVID-19 long-hauler' OR 'chronic COVID syndrome' OR 'long COVID' OR 'post COVID-19 fatigue' OR 'post COVID syndrome' OR 'post COVID condition'**[tw] |
|  | 2 | " **Post-Acute COVID-19 Syndrome**"[mh] |
|  | 3 | **'wearable devices' OR 'wearable sensor' OR 'wearable device' OR 'biosensor' OR 'body sensor network' OR 'bsn' OR 'biomedical sensor' OR 'iot' OR 'wireless wearable technology' OR 'wireless wearable' OR 'wearable electronic devices' OR 'accelerometer' OR 'activity tracker' OR 'pedometer' OR 'sensor' OR 'smartphone'**:[tw] |
|  | 4 | "Wearable Electronic Devices" [mh] |
|  | 5 | **'monitor OR 'physiological monitor*' OR 'vital sign monitor' OR 'monitoring' OR 'disease management'** [tw] |
|  | 6 | "Disease Management" [mh] |
|  | 7 | **#1 OR #2 AND #3 OR #4 AND #5 OR #6**  **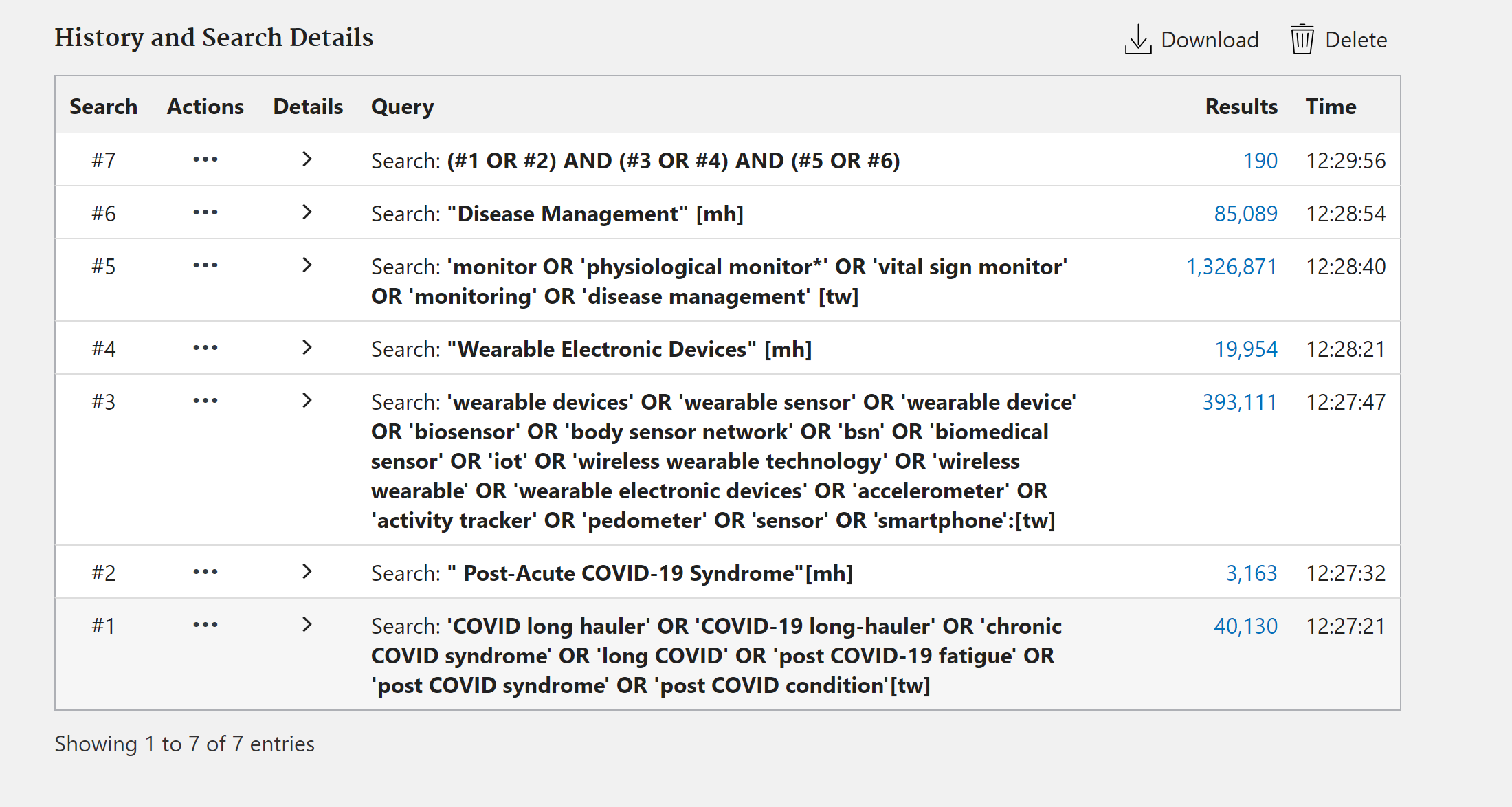** |
| 1. **Cochrane** | 1 | **'COVID long hauler' OR 'COVID-19 long-hauler' OR 'chronic COVID syndrome' OR 'long COVID' OR 'post COVID-19 fatigue' OR 'post COVID syndrome' OR 'post COVID condition'**:ti,kw,ab |
|  | 2 | [mh “**Long COVID**”] |
|  | 3 | **'wearable devices' OR 'wearable sensor' OR 'wearable device' OR 'biosensor' OR 'body sensor network' OR 'bsn' OR 'biomedical sensor' OR 'iot' OR 'wireless wearable technology' OR 'wireless wearable' OR 'wearable electronic devices' OR 'accelerometer' OR 'activity tracker' OR 'pedometer' OR 'sensor' OR 'smartphone'**:ti,kw,ab |
|  | 4 | [mh “Wearable Device”] |
|  | 5 | **'monitor OR 'physiological monitor' OR 'vital sign monitor' OR 'monitoring' OR 'disease management' OR 'symptom management'**:ti,kw,ab |
|  | 6 | [mh "Disease Management"] OR [mh "Monitoring"] OR [mh “symptom”] |
|  | 7 | **#1 OR #2 AND #3 OR #4 AND #5 OR #6**  **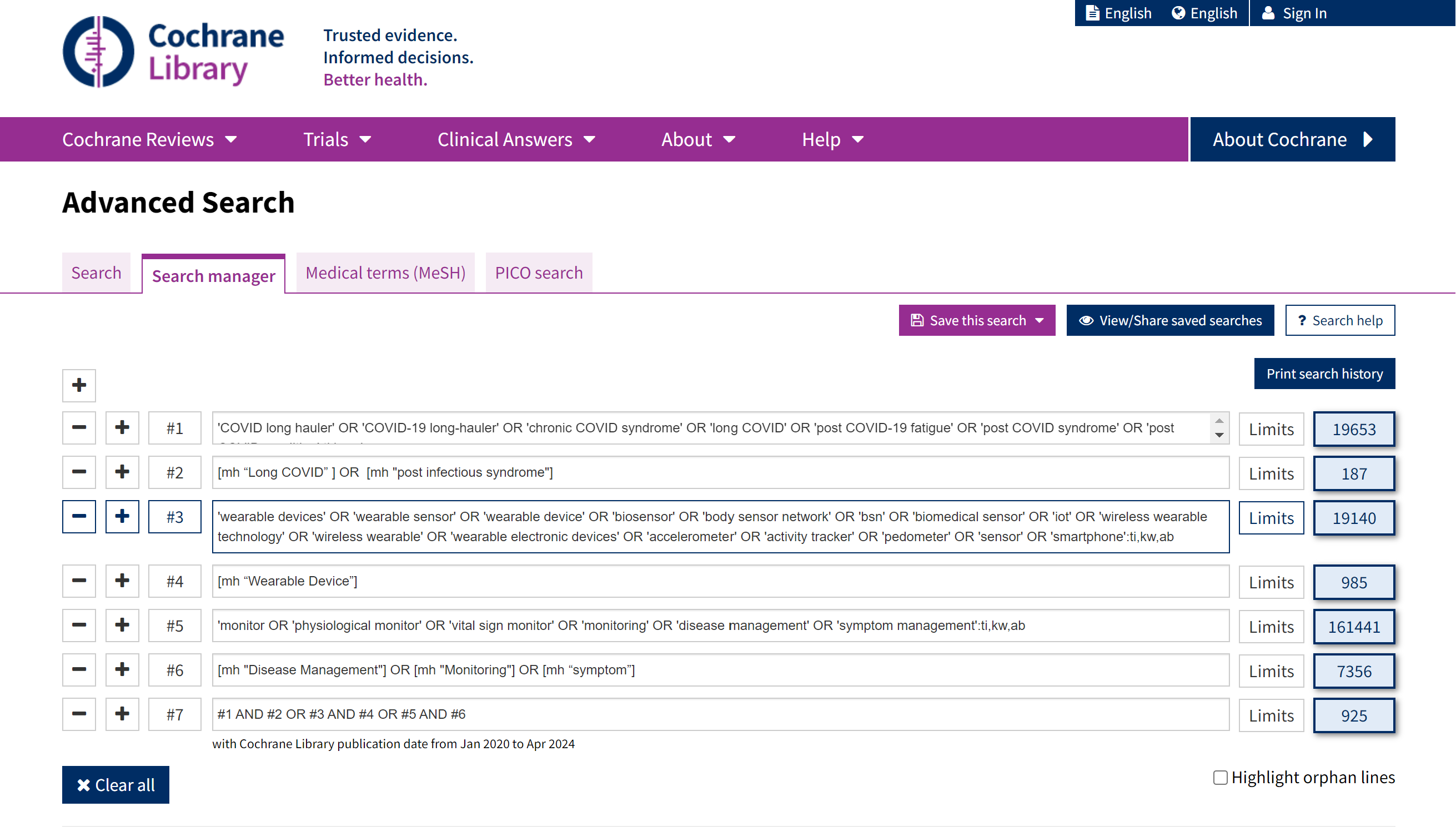** |

**
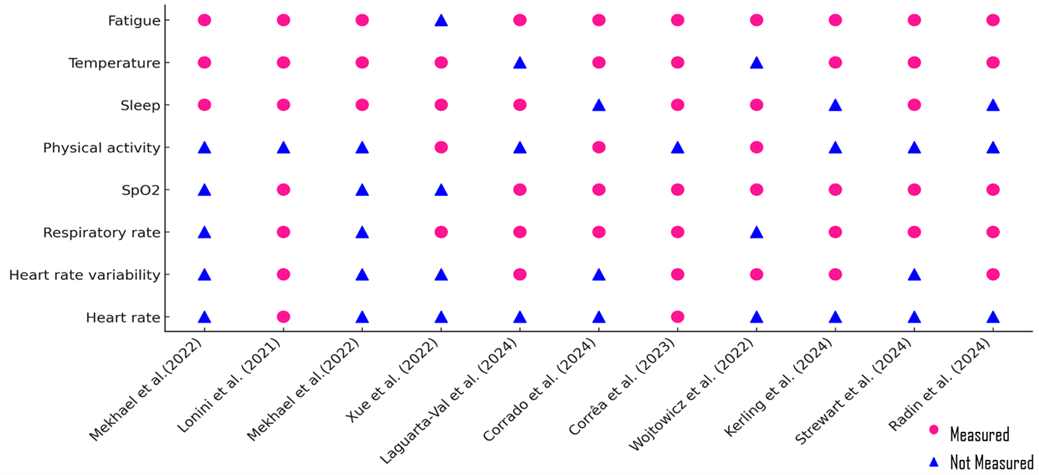
**

**Figure S1:** Showing Long COVID Physiological symptoms measured in the eleven studies included in Scoping review
