# Supplementary material for: Physiological Sensors Equipped in Wearable Devices for Management of Long COVID Persisting Symptoms: Scoping Review
Source: J Med Internet Res. 2025 Mar 26;27:e69506. doi: 10.2196/69506 (PMC11982746; doi:10.2196/69506)
Supplement: Multimedia Appendix 3 [file jmir_v27i1e69506_app3.docx]

| **Sno.** | **Author/objective** | **Wearable device** | **Key outcome** | **Assessment** |
| --- | --- | --- | --- | --- |
| 1 | Mekhael et al.(2022) [5] | Biostrap | Total sleep time | **Duration:** 50 nights of sleep data  **Measure:** The mean measurements for each participant’s total sleep time and sleep phases per night (awake, light, deep, and total) were  calculated and weighted proportionally to the number of days each participant submitted data.  For each participant, the average duration of each sleep phase per night (awake, light, deep, and total sleep time) was calculated.  Formula:  Average Duration =  Total Minutes of Each Phase/Total Number of Nights​ |
| 2 | Lonini et al. (2021) [14] | Suprasternal notch device | Detected altered heart rate, respiration rate, cough frequency and heart rate variability | **Duration:** 1 week throughout a day  **Measure:**  **Respiration**  For each subject, the respiration rate during the pre-walk and post-walk resting periods were calculated using the  accelerometer time series data.  **Cough**  Cough sequences performed and identified as five consecutive, voluntary coughs, were manually clipped and extracted  from the sequence of activities captured in a snapshot. For each sequence, x- and y- axes (200 Hz) were up-sampled to the frequency of the z-axis sampling rate (1600 Hz).  **R-R interval**  The study involved a multi-tier signal filtering approach to extract R-R intervals from the sensor data, including detrending, band-pass filtering, Discrete Wavelet Transform filtering, and threshold-based peak detection algorithms.  **Walking**  To estimate the walking cadence, the walking portion of the sensor recording was manually extracted. |
| 3 | Mekhael et al. (2024) [19] | Biostrap | Motion (44% compliance) | **Duration:** Different time points (2 weeks, 1 month, 6 months, and 12 months)  **Measure:**   - Participants were sent a Biostrap device by mail and were provided with instructions on how to use the device via phone calls and a recorded video detailing the activation steps and linking to the mobile app - Compliance was calculated by dividing the total number of days in which data were transmitted by the total number of days spent in the study |
| 4 | Xue et al. (2022) [15] | Shimmer device, Finapres NOVA | Neurocardiovascular and neuromuscular physiological data in adults with long COVID. | **Duration:**   1. 3-minute lying-to-standing orthostatic test (active stand). 2. 10-minute head-up tilt test following a short participant break. 3. Four 10-second maximum prompted muscle contractions of the thighs ('squeezes') were programmed halfway into rest periods and after both stands.   **Measure:**   - The study measured outcomes by using continuous non-invasive sensing technologies. - The collected data were integrated and visualized to examine the interactions between neurocardiovascular and neuromuscular responses during orthostatic challenges. |
| 5 | Laguarta-Val et al. (2024) [16] | POLAR Ignite 2 device | Nordic walk- PHYSICAL ACTIVITY  . | **Duration:** 45 min Nordic walking sessions once per week for 12 weeks.  **Measure:**  The data collected from the Nordic Walking sessions (distance covered and lactate concentration) were analyzed using two-way ANOVA (group × session; 2 × 12) to compare the effects between the Long-COVID group and the control group across the 12 sessions. This analysis helped determine if there were significant differences in lactate concentration and distance covered between the two groups over time. |
| 6 | Corrado et al. (2024) [17] | Polar H10 chest  Strap/Fitbit Charge 5 smartwatch | HRV-B using diaphragmatic breathing is a feasible intervention for LC. | **Duration:**  Polar H10 Chest Strap: Participants conducted Heart Rate Variability Biofeedback (HRV-B) sessions using the Polar H10 chest strap for 10 minutes, twice daily, over a period of 4 weeks. Fitbit Charge 5: Participants wore the Fitbit Charge 5 smartwatch continuously for a total of 6 weeks. The Fitbit collected nightly HRV data along with other sleep-related measures.  **Measure:**  The Root Mean Square of Successive differences (RMSSD) between heartbeats was a key HRV metric used to assess parasympathetic activity.  The device detected different sleep stages, including light, deep, and REM sleep, by analyzing heart rate and movement data. |
| 7 | Corrêa et al. (2023) [18] | Samsung mobile phone | Static Balance and Mobility | **Duration:**  **Static Balance Assessment:**  Two recordings were performed with eyes open and two with eyes closed. Each recording lasted for 60 seconds. There was a 60-second interval between recordings.  The total time for the static balance evaluation was 7 minutes.  **Mobility Assessment (Instrumented Timed Up and Go (iTUG) Test):**  Participants performed the iTUG test, which involved standing up from a chair, walking along a 3-meter path, making a turn to return to the chair, walking back to the chair, and sitting down.  The duration of the iTUG test was not explicitly mentioned, but it typically lasts for about 10-15 seconds per trial.  **Measure:**  Root Mean Square (RMS) Amplitude: Measures the average magnitude of postural sway in the antero-posterior (AP) and medio-lateral (ML) directions.  Path: The total distance covered by the center of pressure during the balance task, combining both AP and ML displacements.  Time series data from the accelerometer were processed with a zero-lag 10 Hz lowpass filter.  Acceleration values were converted to gravitational units for consistency. |
| 8 | Wojtowicz et al. (2022) [20] | Aidmed | Elevated pulse rate, ECG abnormalities, low oxygen saturation, tachypnea. | **Duration:**  Three times a day for 15 minutes For 10 days.  **Measure:**  Event Count and Duration: Calculated the average duration and standard deviation of detected events (e.g., high pulse, low SpO2, tachypnea).  Event Frequency: Determined the frequency of events per minute.  Patient-Specific Analysis: Individual analysis for each patient, including the number of events and their characteristics, such as duration and threshold values. |
| 9 | Kerling et al. (2024) [6] | Garmin | Total Physical Activity (MET-hours per week) | **Duration:**  45 activity trackers throughout the 3-month period to record daily physical activity and associated parameters such as sleep duration  **Measure:**   1. The highest oxygen uptake over a 30-second interval during the test was considered as V̇O2peak. 2. MET (Metabolic Equivalent of Task): A unit used to estimate the amount of energy expenditure of physical activities. 3. Total Physical Activity: Sum of all physical activities performed by the participant, measured in MET-hours per week. |
| 10. | Strewart et al. (2024) [21] | Fitbit | Tracking physiological changes and predicting long COVID. | **Duration:**  Resting Heart Rate: 24 weeks before to 16 weeks after diagnosis.  Sleep Duration: 24 weeks before to 16 weeks after diagnosis.  Physical Activity: 24 months to 6 months before diagnosis, then continuous monitoring.  **Measure:**  Physiological sensing was continuously tracked using Fitbit wearable devices. These devices provided daily summaries of these metrics without requiring active input from participants. The data were analyzed to compare changes in these physiological signals over time relative to the baseline established before the COVID-19 diagnosis. |
| 11 | Radin et al. (2024) [22] | Fitbit | changes in daily  wearable data (step count, resting heart rate (RHR), and sleep quantity) | **Duration:** 1 year  **Measure:**  Participants’ baseline mean and standard deviation (STD) for RHR, steps, and sleep were calculated using all data collected more than 7 days before symptom onset. Participants had  to have >30 days of baseline data to be included in the sensor comparison. A daily z-score was calculated using the following equation: RHR-score for RHR = (daily mean baseline RHR)/baseline STD RHR.  The mean and 95% confidence interval (CI) of the participant’s daily z score for RHR, steps, and sleep were calculated and compared across different conditions. |
